# The predictive value of sequential cervical length screening in singleton pregnancies after cerclage: a retrospective cohort study

**DOI:** 10.1186/s12884-016-0866-3

**Published:** 2016-04-16

**Authors:** Sophie Pils, Wolfgang Eppel, Regina Promberger, Max-Paul Winter, Rudolf Seemann, Johannes Ott

**Affiliations:** Department of Obstetrics and Gynecology, Medical University of Vienna, Waehringer Guertel 18-20, 1090 Vienna, Austria; Department of Obstetrics and Gynecology, Krankenhaus Hietzing, Vienna, Austria; Department of Internal Medicine II, Medical University of Vienna, Vienna, Austria; Department of Craniomaxillofacial and Oral Surgery, Medical University of Vienna, Vienna, Austria

**Keywords:** Cerclage, Cervical length, Preterm delivery, Delivery, Cervical insufficiency

## Abstract

**Background:**

There are few valid predictors for preterm delivery after cerclage. Experience with a screening program that included four sequential cervical length measurements in singleton pregnancies after cerclage is reviewed.

**Methods:**

In this retrospective cohort study, 88 singleton pregnancies after cerclage were included. Cervical length (CL) measurements were performed perioperatively and at weeks 16 + 0, 18 + 0, 20 + 0, and 22 + 0 by transvaginal ultrasound. Predictive factors for early preterm delivery included patient characteristics, obstetric history and CL measurements and were analyzed separately for women with ultrasound-indicated cerclage and those with history-indicated cerclage. Women with emergency cerclage were excluded.

**Results:**

In women with delivery <35 weeks, CL declined from the 16 + 0 to the 22 + 0 weeks of gestation (*p* = 0.009). In univariate analysis, all CL measurements were predictive for delivery <35 weeks in women who underwent ultrasound-indicated cerclage and in women who received a history-indicated cerclage, whereas in multivariate analysis only CL three to six days after cerclage remained significant (odds ratio 0.85, 95 % CI 0.73–0.98). In women with ultrasound-indicated cerclage, optimized cut-off was ≤20 mm (specificity 83.8 %, sensitivity 84.2 %).

**Conclusions:**

CL measured three to six days after cerclage placement provides the best information about the risk for delivery <35 weeks.

## Background

Preterm birth is a major determinant of fetal outcome and main cause of neonatal morbidity and mortality [1]. Cervical insufficiency is a well-documented etiological factor in preterm delivery (PTD) with an inverse relation between cervical length (CL) and gestational age at delivery [2, 3]. Management strategies for the prevention of PTD include progesterone treatment, vaginal pessaries, and surgical approaches [2, 4]. Although pre- and postconceptional abdominal cerclage has been suggested and evaluated in various studies [5] the main surgical approach is to reinforce the cervix by encircling the bottom half of the endocervical canal and thus compressing it (cerclage) [6]. Depending on indication as well as on characteristics of the pregnancy, women may benefit from cerclage to delay early delivery [1, 7]. From a mechanistically determined point of view, cerclage is assumed to provide structural support to prevent the dynamics of cervical lenght change during rising intrauterine/transfundal pressures [8]. In addition, this procedure might help to maintain at least a mechanical barrier that protects against ascending pathogens [1].

Despite the positive effects on the duration of the pregnancy, there is a lack of valid predictors for PTD after cerclage placement [9]. Fetal fibronectin is also considered a helpful tool to predict upcoming delivery, but the mechanical complications of the cerclage, like displacement, require direct visualization of the cervix and the test is invalid after cervical surgery [9, 10], which makes serial ultrasound examinations the method of choice [11]. However, there is just two reports on serial CL measurements after cerclage [12, 13]. We, thus, aimed to focus on this issue. By studying women after cerclage, we intended to critically review our experience with sequential CL screening which has been implemented at our department. Thus, the main study objective was to evaluate the kinetics of CL in pregnancies after cerclage. To predict early preterm delivery (early PTD) before the 35th gestational week, we also aimed to test the value i) of perioperative CL measurements and basic patient characteristics that would allow an early prediction shortly after the operation, and ii) of sequential measurements.

## Methods

As reported previously [14], a screening program for pregnant women at perceived risk for PTD has been established for many years at the Department of Fetomaternal Medicine of the Medical University of Vienna, Austria. At the department, the annual number of deliveries was at least 2500 during the study period. The department is the national reference center for fetomaternal medicine in eastern Austria. Women with a history of previous PTD due to cervical insufficiency, preterm labor, preterm premature rupture of membranes, a previous 2nd trimester miscarriage or a previous conization were included, as well as women who had undergone cerclage in a current pregnancy. The screening program included CL measurement by transvaginal ultrasound in the 16 + 0, 18 + 0, 20 + 0, and 22 + 0 weeks of gestation. All ultrasound examinations were performed by highly experienced operators (either obstetricians or certified medical-technical assistants). All CL measurements were carried out according to the guidelines of the Fetal Medicine Foundation (available online at http://www.fetalmedicine.com/fmf/training-certification/certificates-of-competence/cervical-assessment/). The shortest of at least three measurements was documented.

From June 2000 to December 2012, a total of 222 cerclage procedures were performed. In this retrospective analysis, we included women with (i) a singleton pregnancy, (ii) a history of previous PTD (i.e. 22 + 0-36 + 6) or 2nd trimester miscarriage who (iii) underwent cerclage in the current pregnancy. The study population had regular follow-up examinations, beginning with first-trimester screening, and must have given birth at the department from January 2001–July 2013 (*n* = 88). Notably, according to the local guidelines, which is based on previous reports [15, 16] for women at risk for PTD, women were offered an ultrasound-indicated cerclage if the CL was <25 mm which was the case for 56/88 women (63.6 %). However, 32 women (36.4 %) underwent the procedure on their own demand regardless of CL. They insisted on being treated with a history-indicated cerclage due to their poor obstetric history. At the 16 + 0, 18 + 0 and 20 + 0 week, 52 (59.1 %), 76 (86.4 %) and 88 (100.0 %) had undergone cerclage, respectively.

We excluded women with multiple pregnancies (*n* = 32), women with emergency cerclage (*n* = 30), those who had to be delivered electively preterm for maternal-fetal complications (*n* = 39) and those who delivered at another department and, thus, were lost to follow-up (*n* = 33). This resulted in a patient population of 88 women after cerclage. None of the women had been treated with prophylactic progesterone.

The main outcome measure was early PTD before 35 week of gestation in accordance with recent studies [11, 14]. This also included cases of second trimester miscarriage due to cervical insufficiency (defined as painless cervical dilatation leading to second-trimester birth), preterm labor, or preterm premature rupture of membranes. This study was approved by the Institutional Review Board of the Medical University of Vienna (IRB number: 1730/2013).

Retrospective acquisition of all relevant data was performed using the Viewpoint® software (GE Healthcare, Wessling, Germany) which is the basic perinatologic database at the department. CL measurements were done one day before and after a median of 4 days (IQR 3-6) after cerclage as well as at weeks 16 + 0, 18 + 0, 20 + 0 and 22 + 0. In addition, the following parameters were included: history of previous conization procedures, age at delivery, body mass index (BMI) at the initial visit, parity, previous preterm birth due to cervical insufficiency, preterm labor, or preterm premature rupture of membranes, previous 2nd trimester miscarriage, pregnancies after in-vitro-fertilization (IVF), urinary tract infection during pregnancy, and cigarette smoking.

All women in the cohort were treated with the modified Shirodkar technique. Accordingly, a 5 mm Mersilene® Polyester Fiber Suture (Ethicon Inc., Somerville, New Jersey, USA) was passed anteriorly to posteriorly on the cervix. The tape was tied anteriorly, and the cervical mucosa was then closed with continuous stitches [17].

Neither written nor verbal informed consent is necessary in retrospective studies according to the Ethics Committee of the Medical University of Vienna and was, thus, not obtained.

### Statistical analysis

Nominal variables are reported as numbers and frequencies, and continuous variables as medians and interquartile ranges (IQR). Paired t-tests were applied to test for differences between subsequent CL measurements within one group. An unpaired *t*-test was used to test for the differences in initial CL between women with and without early PTD For t-tests, data had to be normally distributed as evaluated by Kolmogorow-Smirnow-Test. This was the case for all of these analyses. For t-tests, the t-value and the degree of freedom (dg) are provided. In a stepwise linear regression model for prediction of early PTD, we included the following parameters in addition to baseline patient characteristics: i) basic patient characteristics, ii) the CL before and after cerclage, the dynamics between these measurements, and iii) the CL at the completed 20 and 22 weeks of gestation. These analyses were performed to allow an early prediction of early PTD shortly after cerclage. Women with history-indicated and ultrasound-indicated cerclage were analysed separately. The optimal cut-off for CL was calculated as the threshold value with the highest specificity and sensitivity based on the receiver-operating characteristics (ROC) curve as a sensitivity versus (1 − specificity) plot. The discriminatory ability of the investigated parameters is described as the correlation between specificity and sensitivity, and was measured by the area under the receiver-operating (AUC) curve. Where appropriate, values are given with a 95 % confidence interval (95 % CI). Statistical analysis was performed using the open-source statistical package, R (version 2.13.0; available online at http://www.r-project.org/). Differences were considered statistically significant if *p* < 0.05.

## Results

Of the cases, 20 (22.7 %) delivered at <35 weeks of gestation. In addition, four women (4.5 %) suffered from a second trimester miscarriage (22–24 weeks). This resulted in an overall rate for early PTD <35 weeks used for the following analyses of 27.3 % (24/88). Details on patient characteristics are shown in Table 1. This Table also provides a comparison to the 33 women lost to follow-up.

**Table 1:** Basic patient characteristics: comparison of the analysed study population and patients lost to follow-up

		Analzyed patients	Lost to follow-up	*p*
		(*n* = 88)	(*n* = 33)	
Age (years)^c^		31 (29;36)	32 (27;38)	0.828
Body mass index (kg/m^2^)^c^		24.1 (20.9; 28.5)	22.2 (21.1;26.4)	0.604
Previous preterm delivery^d^		40 (45.5)	15 (45.5)	1.000
Previous second trimester miscarriage^d^		59 (67.0)	20 (60.6)	0.508
Previous conization^d^		15 (17.0)	4 (12.1)	0.587
Pregnancy after IVF treatment^d^		5 (5.7)	4 (12.1)	0.254
Urinary tract infection during pregnancy^d^		2 (2.3)	3 (9.1)	0.124
Parity	0	19 (21.6)	8 (24.2)	0.932
	1	31 (35.2)	11 (33.3)	
	≥2	38 (43.2)	14 (42.4)	
Cigarette smoking^d^		15 (17.0)	3 (9.1)	0.392
Gestational age at cerclage^c^		16 (16;18)	16 (16;18)	0.496

**Fig. 1:**
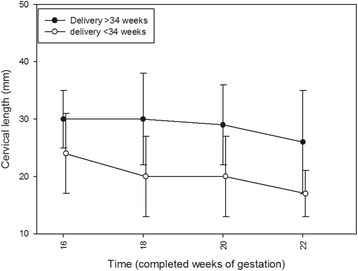
Dynamics in cervical lengths in the course of routine screening in women with (n = 24, white dots) and without early preterm delivery (*n* = 64, black dots). All women - regardless of cerclage indication - are included. The group of women with early preterm delivery includes those with a second trimester miscarriage. Gestational age is plotted on the x-axis

Details about changes in CL are provided in Fig. 1. CL at 16 weeks gestation did not differ between women with and without previous early PTD (*n* = 64; median 30 mm, IQR 23–38 vs. *n* = 24; median 24 mm, IQR 17–34; *p* = 0.953 with *t* = 1.357 and df = 87 in unpaired *t*-test, respectively). When comparing week 16 + 0 to weeks 18 + 0 and 20 + 0, significant shortening in CL was found in women with early PTD (*p* = 0.026 with *t* = 2.423 and df = 47 and *p* = 0.009 with *t* = 2.930 and df = 47 in paired *t*-test, respectively), whereas in women without early preterm delivery, there were no significant differences (*p* = 0.816 with *t* = -0.234 and df = 127 and *p* = 0.088 with *t* = 1.736 and df = 127 in paired *t*-test, respectively).

For both women who had undergone ultrasound-indicated cerclage (early PTD: 19/56, 33.9 %; Table 2) and women who had undergone history-indicated cerclage (early PTD: 5/32, 15.6 %; Table 3), the logistic regression models’ univariate analyses revealed that all perioperative CL parameters differed significantly between women with and without early PTD. In multivariate analyses, all parameters that had been significant in the univariate models were included apart from the parameter “decrease in CL after cerclage”, due to its co-linear association (t-value 9.267, *p* < 0.001) with the parameters “CL before cerclage” and “CL after cerclage,” which were also more predictive univariately. Notably, only CL after cerclage remained significant (ultrasound-indicated: OR 0.85, 95 % CI: 0.73–0.98; *p* = 0.022; history-indicated: OR 0.85, 95 % CI: 0.75–0.96; *p* = 0.004).

**Table 2:** Prediction of early preterm delivery in women who had undergone cerclage for cervical shortening. Results of the univariate and multivariate analysis

				Univariate analysis			Multivariate analysis		
		Delivery <35 week (*n* = 19)	Delivery ≥35 week (*n* = 37)	OR (95 % CI)^a^	P	P	Adjusted OR (95 % CI) ^a^	P	P
					(Wald’s test)	(LR test)^b^		(Wald’s test)	(LR test)^b^
Age (years)^c^		33 (27;38)	31 (29;36)	1.01 (0.92,1.12)	0.758	0.757	-	-	-
Body mass index (kg/m^2^)^c^		24.8 (21.0; 30.4)	23.2 (20.2;27.2)	1.09 (0.96,1.23)	0.189	0.183	-	-	-
Previous preterm delivery^d^		7 (36.8)	22 (59.5)	0.4 (0.13,1.24)	0.113	0.107	-	-	-
Previous second trimester miscarriage^d^		13 (68.4)	20 (54.1)	1.84 (0.58,5.9)	0.304	0.297	-	-	-
Previous conization^d^		3 (15.8)	9 (24.3)	0.33 (0.09,1.21)	0.094	0.072	-	-	-
Pregnancy after IVF treatment^d^		3 (15.8)	2 (5.4)	3.28 (0.5,21.6)	0.217	0.211	-	-	-
Urinary tract infection during pregnancy^d^		0 (0)	1 (2.7)	0 (0,Inf)	0.992	0.360	-	-	-
Parity^c^	0	8 (42.1)	4 (10.8)	reference	reference	0.292	-	-	-
	1	6 (31.6)	15 (40.5)	0.33 (0.02;5.03)	0.427		-	-	-
	≥2	5 (26.3)	18 (48.6)	0 (0;inf)	0.996		-	-	-
Cigarette smoking^d^		3 (15.8)	7 (18.9)	0.8 (0.18,3.54)	0.772	0.770	-	-	-
Gestational age at cerclage^c^		16 (16;18)	16 (16;18)	1.14 (0.66,1.96)	0.634	0.633	-	-	-
CL^e^ before cerclage (mm)^c^		17 (14;20)	23 (17;24)	0.89 (0.8,0.99)	*0.036* ^*f*^	*0.028* ^*f*^	0.98 (0.86,1.13)	0.808	0.809
CL^e^ after cerclage (mm)^c^		14 (9;19)	27 (22;32)	0.82 (0.74,0.91)	*<0.001* ^*f*^	*<0.001* ^*f*^	0.85 (0.73,0.99)	0.041	*0.024* ^*f*^
Decrease in CL^e^ after cerclage^d^		13 (68.4)	7 (18.9)	9.29 (2.61,33.06)	*<0.001* ^*f*^	*<0.001* ^*f*^	Not included due to redundancy		
CL^e^ at the 20 gestational week (mm)^c^		16 (10;23)	24 (21;31)	0.84 (0.76,0.93)	0.001	*<0.001* ^*f*^	0.98 (0.82,1.16)	0.803	0.803
CL^e^ at the 22 gestational week (mm)^c^		15 (5;23)	20 (16;29)	0.89 (0.83;0.97)	0.007	*0.002* ^*f*^	0.97 (0.87,1.08)	0.595	0.593

^a^ OR (95 % CI) = odds ratio (95 % confidence interval), ^b^ LR test = likelihood ratio test, ^c^ Continuous variable, provided in median (interquartile range); ^d^ nominal variable, provided in n (%); ^e^ CL = cervical length; ^f^ Italic letters indicate statistical significance; ^g^ despite its significant predictive value in the univariate analysis, the parameter “cervical length before cerclage” was not included in the multivariate model due to its co-linear association with the parameter “cervical length after cerclage” that was more predictive in the univariate analysis

**Table 3:** Prediction of early preterm delivery in women who had undergone cerclage regardless of cervical length. Results of the univariate and multivariate analysis

				Univariate analysis			Multivariate analysis		
		Delivery <35 week (*n* = 5)	Delivery ≥35 week (*n* = 27)	OR (95 % CI)^a^	P	P	Adjusted OR (95 % CI) ^a^	P	P
					(Wald’s test)	(LR test)^b^		(Wald’s test)	(LR test)^b^
Age (years)^c^		31 (29;39)	31 (29;35)	1.09 (0.89;1.34)	0.413	0.401	-	-	-
Body mass index (kg/m^2^)^c^		23.2 (19.6;24.4)	25.7 (21.4;29.4)	0.84 (0.65;1.09)	0.191	0.124	-	-	-
Previous preterm delivery^d^		2 (40.0)	9 (33.3)	1.33 (0.19;9.47)	0.774	0.775	-	-	-
Previous second trimester miscarriage^d^		4 (80)	22 (81.5)	1.27 (0.10;34.76)	1.000	1.000	-	-	-
Previous conization^d^		1 (20.0)	2 (7.4)	3.12 (0.23,43.02)	0.394	0.420	-	-	-
Pregnancy after IVF treatment^d^		0	0	-	-	-	-	-	-
Urinary tract infection during pregnancy^d^		0	1 (3.7)	0 (0;inf)	0.995	0.557	-	-	-
Parity^d^	0	2 (40.0)	5 (18.5)	0.63 (0.19,2.06)	0.447	0.583	-	-	-
	1	1 (20.0)	9 (33.3)	0.28 (0.02;3.88)	0.341		-	-	-
	≥2	2 (40.0)	13 (48.2)	0.38 (0.04;3.52)	0.398		-	-	-
Cigarette smoking^d^		1 (20.0)	4 (14.8)	1.44 (0.13;16.41)	0.770	0.750	-	-	-
Gestational age at cerclage^c^		15 (14;18)	16 (14;17)	1.02 (0.58;1.82)	0.941	0.942	-	-	-
CL^e^ before cerclage (mm)^c^		33 (31;35)	37 (34;40)	0.82 (0.65;1.04)	0.098	*0.066*	1.06 (0.95,1.19)	0.283	0.260
CL^e^ after cerclage (mm)^c^		30 (19;33)	42 (35;47)	0.72 (0.51;1.01)	0.055	*<0.001* ^*f*^	0.85 (0.75;0.96)	0.007	*0.004* ^*f*^
Decrease in CL^e^ after cerclage^d^		4 (80.0)	5 (18.5)	17.6 (1.60;193.34)	0.019	*0.008* ^*f*^	Not included due to redundancy		
CL^e^ at the 20 gestational week (mm)^c^		30 (19;31)	40 (29;45)	0.91 (0.83;1.01)	0.069	*0.046* ^*f*^	1.01 (0.90:1.14)	0.875	0.876
CL^e^ at the 22 gestational week (mm)^c^		19 (12;25)	38 (26;43)	0.89 (0.79;0.99)	0.049	*0.019* ^*f*^	0.94 (0.86;1.03)	0.170	0.157

^a^ OR (95 % CI) = odds ratio (95 % confidence interval), ^b^ LR test = likelihood ratio test, ^c^ Continuous variable, provided in median (interquartile range); ^d^ nominal variable, provided in n (%); ^e^ CL = cervical length; ^f^ Italic letters indicate statistical significance; ^g^ despite its significant predictive value in the univariate analysis, the parameter “cervical length before cerclage” was not included in the multivariate model due to its co-linear association with the parameter “cervical length after cerclage” that was more predictive in the univariate analysis

In women after ultrasound-indicated cerclage, the optimized cut-off for the prediction of early preterm delivery was a CL three to six days after cerclage ≤20 mm (area under the curve: 0.867) which resulted in specificity, sensitivity, and positive and negative predictive values of 83.8 % (95 % CI: 61.9–93.7), 84.2 % (95 % CI: 60.4–96.6), 72.7 % (95 % CI: 49.8–89.3), and 91.2 % (95 % CI: 76.3–98.1), respectively. Of the 22 women with a CL ≤20 mm, 16 (72.7 %) had early PTD compared to 3/34 (8.8 %) women with a CL >20 mm (*p* < 0.001).

In women after history-indicated cerclage, the optimized cut-off was ≤33 mm (area under the curve: 0.915) which resulted in specificity, sensitivity, and positive and negative predictive values of 83.3 % (95 % CI: 61.9–93.7), 100.0 % (95 % CI: 40.3–100.0), 55.5 % (95 % CI: 18.7–81.3), and 100.0 % (95 % CI: 80.7–100.0), respectively. Four/10 (40 %) women with a CL ≤33 mm had early PTD compared to 1/12 (8.3 %) women with CL >33 mm (*p* = 0.135).

## Discussion

This retrospective study on singleton pregnancies after cerclage provided the following key findings: i) in cases that resulted in an early PTD, CL shortened significantly from week 16 to measurements obtained at time points thereafter; ii) the absolute CL three to six days after cerclage was the most predictive parameter for early PTD regardless from the indication for cerclage; and iii) for this specific measurement, a threshold of ≤20 mm gives the most accurate information about the risk for early PTD after cerclage in women who had undergone ultrasound-indicated cerclage.

Besides the small sample size, we consider the retrospective design of our study as a limitation, since it might have introduced bias. For example, a selection bias is possible, since we cannot prove that all women at perceived risk have undergone the screening program. Unfortunately, 33 women were lost to follow-up. We consider this circumstance of minor relevance, since, as demonstrated in Table 1, patient characteristics did not differ between analyzed women and those lost to follow-up. Moreover, the lack of various other parameters that have been suggested as predictive of the duration of pregnancy after cerclage needs to be emphasized. A previous report claimed that shortening CL at week 26 was highly predictive for early PTD [10]. Since we aimed to evaluate early predictive parameters, we did not focus on CL at this gestational age. Furthermore, we cannot comment on cervical funneling [12, 18, 19]. This information could not be reliably evaluated by retrospective chart review. Neither can we provide details on cerclage height [20].

It has to be mentioned that one could be concerned about the terms “ultrasound-indicated” and “history-indicated” cerclage which was based on a Cochrane database review [16]. However, the latter could also be named “elective” as used in a recent report [13]. We hope that the exact definitions provided in the Methods Section will make replicability and comparability to other studies possible.

The mechanism of action of cerclage is poorly understood, a mechanical component has been suggested [8]. This assumption is supported by CL shortening in women with early PTD (Fig. 1). Similar prospective data have already been reported [21] and the authors concluded that serial CL measurements in the late second or early third trimester could be used as an early warning tool. This report shares another similarity with our results: The investigators found no association between the difference in CL before and after cerclage and pregnancy outcome [21]. We could partly confirm these observations, at least after multivariate analysis of possible predictive parameters (Tables 2 and 3).

Significant direct correlations between the pre- and post-cerclage CL with pregnancy duration have already been reported previously [12]. However, it is noteworthy that in our study, only the CL after cerclage achieved independent significance for early PTD after multivariate analysis, whereas CL before cerclage did not. Nevertheless, this observation might be effect of the small sample size. However, in the multivariate analysis in women with ultrasound-indicated cerclage, it becomes evident that the adjusted odds ratios of the non-significant parameters are about one, which is in line with the high p-values. This suggests that the lack of significance for these parameters is consistent. Accordingly, a change from the pre- to the postoperative CL would have an influence on the duration of pregnancy, but this information would be less important than the absolute CL that is achieved by the operation. Focusing on postoperative CL as a single factor in women who underwent ultrasound-indicated cerclage, a cut-off of 20 mm seems to be optimal to assess the risk for early PTD, which is quite comparable to previous results [22]. Since cerclage was indicated in cases with CL <25 mm, we do not consider it surprising that the optimized predictive CL after surgery (20 mm) was similar and that this fits previous findings.

According to our model, whether the operation would result in an increase or a decrease in CL would be of only minor relevance for the final assessment of a woman’s prognosis. This phenomenon is described for the first time. Taking into account that significant cervical shortening was found only in women with early PTD (Fig. 1), it is reasonable that women with a short CL after cerclage - the main predictive parameter - are more prone to subsequent cervical shortening. Hypothetically, this process might depend on a “peak CL” after cerclage.

Consequently, women with a short CL might benefit from the procedure only if an increase in length was achieved, and women with a long preoperative CL value might not benefit from it. As reported previously, in women with a CL <25 mm, the procedure would decrease the chance for early PTD by 30 % [23]. Thus, cerclage has been emphasized to be restricted to the minority of women with a short CL [15]. Notably, 38 % of women underwent history-indicated cerclage, on their own demand. This included women who received an early intervention before the 16 week despite a CL ≥25 mm. Accordingly, Fig. 1 shows a median CL value in women without early preterm delivery of about 30 mm in week 16. It needs to be emphasized that some women with a CL >25 mm might have received a cerclage later on due to shortening of the cervix. We, thus, believe that most of the beneficial outcome of cerclage in the history-indicated group was due to this assumed subset of patients.

## Conclusions

CL measured three to six days after cerclage provides the best predictive information when compared with other perioperative parameters. After ultrasound-indicated cerclage, the optimal cut-off to assess the risk for early PTD is 20 mm. The significant CL shortening in women with early PTD suggests that a mechanical mechanism of action contributes to the effect of cerclage. Although our study sheds new light on the issue of CL before and after cerclage, prospective, larger studies are needed to confirm our results.

## Abbreviations

CL: cervical length
IQR: interquartile ranges
PTD: preterm delivery
early PTD: early preterm delivery
ROC: receiver-operating characteristics
